# The Ecophysiological Response of Olive Trees under Different Fruit Loads

**DOI:** 10.3390/life14010128

**Published:** 2024-01-16

**Authors:** Efthymios Kokkotos, Anastasios Zotos, Angelos Patakas

**Affiliations:** 1 Laboratory of Plant Production, Department of Food Science and Technology, University of Patras, 30100 Agrinio, Greece; ekokkotos@upatras.gr; 2 Department of Sustainable Agriculture, University of Patras, 30100 Agrinio, Greece; azotos@upatras.gr

**Keywords:** sink–source effect, alternate bearing, thermocouple psychrometry, non-structural carbohydrates, carbon-stable isotope ratio

## Abstract

Olive trees have a unique reproductive pattern marked by biennial fruiting. This study examined the repercussions of alternate fruit bearing on the water relations of olive trees and the associated ecophysiological mechanisms. The experiment spanned two consecutive years: the “ON” year, characterized by a high crop load, and the “OFF” year, marked by minimal fruit production. Key ecophysiological parameters, including sap flow, stomatal conductance, and photosynthetic rate, were monitored in both years. Pre-dawn water potential was measured using continuous stem psychrometers and the pressure chamber technique. Biochemical analyses focused on non-structural carbohydrate concentrations (starch, sucrose, and mannitol) and olive leaves’ carbon-stable isotope ratio (δ^13^C). Results revealed a higher leaf gas exchange rate during the “ON” year, leading to an average 29.3% increase in water consumption and a 40.78% rise in the photosynthetic rate. Higher water usage during the “ON” year resulted in significantly lower (43.22% on average) leaf water potential. Sucrose and starch concentrations were also increased in the “ON” year, while there were no significant differences in mannitol concentration. Regarding the carbon-stable isotope ratio, leaves from the “OFF” year exhibited significantly higher δ^13^C values, suggesting a higher resistance to the CO_2_ pathway from the atmosphere to carboxylation sites compared to the “ON” year plants.

## 1. Introduction

The olive (*Olea europaea* L.) is one of the most important fruit crops worldwide, with a significant contribution to the economy of many Mediterranean countries [1]. Olive trees have a unique reproductive habit, with alternate bearing being a common phenomenon [2]. Alternate bearing refers to the tendency of a fruit tree to produce a full crop load in one year (the “ON” year) followed by a low crop load in the following year (the “OFF” year), which can significantly affect tree productivity. The vegetative development is restricted during the “ON” year, which impacts flower induction and, consequently, the following year’s production. The competitive dominance of growing fruits on shoot meristems regarding the photosynthates is responsible for this decrease in vegetative development [3], generally referred to as the “sink–source effect”.

The sink–source theory is a widely accepted conceptual framework for understanding the relationship between fruit production and plant growth [4]. According to this theory, the plant is divided into two major components: the source, which refers to the leaves and other photosynthetic organs that produce carbohydrates through photosynthesis, and the sink, which refers to the fruit and other non-photosynthetic organs, such as roots, that consume carbohydrates for growth and development. The balance between source and sink determines the rate of photosynthesis and the allocation of resources within the plant. Particularly heavy crop loads are reported to increase stomatal conductance and, therefore, the photosynthetic rate in various species like apples [5,6,7], avocados [8], peaches [9,10], citrus [11,12], grapevines [13,14,15], and olives [16,17,18,19]. Two primary mechanisms are reported to be involved in the downregulation of photosynthesis as a result of alternations in the sink–source balance; one is by decreasing Rubisco content or its activation state [20], and the other is by reducing stomatal and mesophyll conductance, which could lead to a reduction in CO_2_ concentration in the sites of carboxylation, potentially as a result of the accumulation of carbohydrates [21]. Indeed, Bustan et al. [22] found that during summer, when there was a high demand for carbohydrates for fruit growth and oil production, the stored non-structural carbohydrates in olives during the “ON” years decreased. Moreover, Haouari et al. [16] reported an accumulation of leaf soluble sugars and starch concentrations after severe fruit thinning in *Olea europaea* cv. *Besbassi*. Mainly, sucrose and mannitol are considered the dominant soluble sugars and, along with starch, consist of the most abundant carbohydrates in the leaves of olive trees [22,23]. Additionally, owing to the abundance of these two sugars, they have also been deemed the primary translocated sugars in the leaves and branches of olive trees [24]. On the other hand, alterations in stomatal conductance are known to be closely related to plant water status. In particular, high crop load is reported to result in lower stem water potential values in several species, such as apples [25,26], nectarines [27], and peaches [28]. In contrast, other researchers reported no effect on plums [29] and peaches [30]. However, regarding olive trees, not only has little research been conducted on the relationship between fruit load and tree water status, but contradictory results have also been reported. Specifically, while Naor et al. [31] and Bustan et al. [32] found that high values of the sink–source ratio led to a decrease in stem water potential, Gucci et al. [33] reported no such effect. On the other hand, Trentacoste et al. [34] observed a significant effect when Ψ_stem_ dropped below −1.4 MPa. This contradiction in reported results can be partly attributed to the well-known methodological difficulties in obtaining continuous water potential data using the pressure chamber technique [35,36]. However, implementing recently developed and automated technologies such as thermocouple psychrometry allows for the collection of robust data sets after proper calibration, thus facilitating the continuous monitoring of plant water status.

Thus, the aims of this study are to evaluate the effects of alternate fruit bearing in olive trees’ physiological and hydrodynamic parameters and to identify the ecological significance and mechanisms underlying alternate bearing impact in olive trees.

## 2. Materials and Methods

### 2.1. Experimental Orchard

The study was carried out in a commercial olive orchard in Aitoloakarnania prefecture, Western Greece, which was planted with the “Kalamon” olive variety (*Olea europea* cv. “*Kalamon*”). The region has a typical Mediterranean climate with mild, rainy winters and hot, dry summers. The mature olive trees were planted in a 7 m × 7 m formation (ca. 204 trees per ha) in rows oriented north-northeast to south-southwest. These trees were cultivated in heavy clay soil with adequate depth and were irrigated using a sprinkler system, with one sprinkler per tree, providing a flow rate of 320 L per hour, according to the irrigation strategy commonly used by the farmers of this area. The same agricultural practices, including fertilization and plant protection, were followed in both study years to maintain the trees in excellent phytosanitary condition and with the full availability of nutrients. Additionally, the trees were not pruned from the beginning of the growing season in 2021 until the period prior to the harvest of the 2022 season.

### 2.2. Olive Bearing Cycle

The study was carried out for two consecutive years, with 2021 designated as the “OFF” year, characterized by low fruit production, while the other year (2022) was designated as the “ON” year, with high yields. During the “ON” year, the olive orchard produced a typical full crop load for the specific cultivar and orchard of 20.4 tons per hectare. On the other hand, during the “OFF” year, a low yield of 3.43 tons per hectare was recorded, indicating low production.

### 2.3. Monitoring of Soil Water Content and Meteorological Parameters

Throughout the experiment, a weather station permanently installed in the field was used to continuously monitor various microclimatic parameters. Hourly measurements of solar radiation (Wh/m^2^), wind speed at a height of 2 m (km/h), rainfall (mm), air relative humidity (%), and air temperature (°C) were collected and used to calculate the potential evapotranspiration (ETo) according to the Penman–Monteith equation [37].

Additionally, soil water content was continuously measured using capacitance sensors (EnviroSCAN, Sentek Sensor Technologies, Stepney, Australia) at a depth of 100 cm. To ensure accurate measurements, the sensors were calibrated according to the manufacturer’s instructions and installed in a manner that covered the greatest absorption root area [38,39,40] with measurements taken at 10 cm intervals.

### 2.4. Measurement of Ecophysiological and Hydrodynamic Parameters

Stem water potential (Ψ_stem_) values were obtained every 30 min using thermocouple psychrometers (PSY1, ICT International, Armidale NSW, Australia) installed on one main branch in two trees. The psychrometers’ calibration and installation followed the manufacturer’s recommended procedure. Initially, to calibrate the stem psychrometers, a series of standard sodium chloride (NaCl) solutions with known molality ranging from 0.1 M to 1.0 M were prepared. These concentrations correspond to the range of water potentials that plants typically experience, according to Lang [41]. Regarding the installation, a flat spot was initially engraved on the branch allowing the proper contact of the thermocouples with the sample surface. Following this, they were fully sealed with silicon grease and insulated with successive layering of foam materials and reflective coating to avoid thermal gradients due to incident sunlight.

Moreover, since there are not adequate results in the literature concerning the accuracy of this methodology in olive trees, concurrent measurements of water potential were also conducted during pre-dawn with a pressure chamber bomb (SKPM 1400/80, Skye Instruments, Powys, UK). For this measurement, five apical stems per tree with ca. five leaf pairs per stem were used [42].

Sap flow estimations were made using the heat ratio methodology (HRM-SFM1 Sap Flow Meter, ICT International, Armidale NSW, Australia). This technique was preferred because of its precision in measuring low sap velocity and its ability to provide robust measurements in olive tree water relations studies [43]. Briefly, this methodology is used to measure sap flow velocity by estimating the ratio of temperature increase following the emission of a short heat pulse [44]. Specifically, four sets of sensors were installed at an azimuthal angle of 90° and 50 cm above the soil surface on two selected trees, located in the center of the experimental orchard, during both study years. Using more sensors per tree instead of more sample trees with fewer sensors per tree was considered the most appropriate method for obtaining reliable results, since the azimuthal variability of sap flow is known to be considerably high in mature olive trees [45]; thus, it must be thoroughly assessed by a sufficient number of sensors [46] in order to increase the accuracy of the results. On the other hand, sap flow values appeared not to be significantly different between plants in the same orchard, a fact that is consistent with the results of Moreno et al. [47], Fuentes et al. [48], López-Bernal et al. [49], and Hernandez-Santana et al. [50], who also performed similar experiments using the same number of sample trees.

Prior to installation, to identify the active xylem in the sapwood area, a sapwood core sample was extracted from the trunks of both trees using a tree-coring tool. Subsequently, methyl orange dye was used to differentiate the sapwood from the heartwood by applying it to the extracted sample with a micropipette. The active conducting xylem was then isolated, and its depth was measured using calipers. The sap flow measurements were used to estimate tree water consumption, and to compare it between the two study years, the ratio of daily sap flow to daily potential evapotranspiration (hereafter normalized sap flow, SF_norm_) was calculated.

Maximum values of both stomatal conductance and net photosynthetic rate were measured on ten healthy, fully matured exterior leaves using an open gas analyzer system (LCPro+, ADC, Bioscientific Ltd., Hoddesdon, UK) under light-saturated conditions (i.e., photosynthetically active radiation at leaf surface higher than 1200 μmol/m^2^s) from 9:00 am to 11:00 a.m., on the same days when water potential was measured with the pressure chamber bomb.

### 2.5. Leaf Area Measurements

Leaf area index (LAI) was measured using the LAI-2000 Plant Canopy Analyzer (LI-COR Biosciences, Lincoln, NE, USA) as described by Villalobos et al. [51] on the same days as water potential and stomatal conductance measurements [52]. LAI_max_ was measured at 50–80 cm from the trunk and LAI_min_ at the center of the canopy since all plants had an open-shape canopy through pruning. LAI_avg_ was calculated by integrating plant ground cover (GC) using the following equation [53]:

$${\mathrm{LAI}}_{\mathrm{avg}}={\mathrm{LAI}}_{\max}*\mathrm{GC}+{\mathrm{LAI}}_{\min}*\left(1-\mathrm{GC}\right)$$

### 2.6. Measurement of Soluble Sugars and Starch Concentration

Analytical measurements regarding the concentration of non-structural carbohydrates in olive tree leaves were conducted using a Dionex P680 high-performance liquid chromatography (HPLC) system (Dionex Corporation, Sunnyvale, CA, USA) [54] on ten fully mature leaves (five from each tree) which were collected on DOY 210, 224, 243, and 264. The samples were immediately frozen in liquid nitrogen, kept at −80 °C, and subjected to freeze-drying. After recording their weight, the samples were grounded into a fine powder with a mill (pulverisette 11, Fritsch GmbH 93 Milling and Sizing, Idar-Oberstein, Germany). Soluble sugars were extracted from 100 mg of fine leaf powder with 5 mL of ethanol (80%). The tubes were maintained in a heating block at 80 °C for 1 h and periodically shacked gently. Then, they were centrifuged, and supernatants were used to perform the measurements in the mobile phase, which consisted of degassed, distilled, and deionized water at a 0.6 mL/min flow rate. This process was repeated twice. Glucose extraction from starch was used to measure starch concentration according to the amyloglucosidase digestion (A-3042) procedure described by Schaffer et al. [55]. Sucrose, mannitol, and starch concentrations were all expressed in mg/gr of dry weight.

### 2.7. Measurement of the Carbon-Stable Isotope Ratio

The contribution of different CO_2_ resistances from the atmosphere to the sites of carboxylation in chloroplasts was evaluated by determining the carbon-stable isotope ratio in the leaves of both treatments using an Elementar Isoprime 100 isotope ratio mass spectrometer (IRMS) (IsoPrime Ltd., Cheadle Hulme, UK) coupled to an elemental analyzer (Elementar Vario Isotope EL Cube, Elementar Analysensystem GmbH, Hanau, Germany). Measurements were performed in twenty (20) leaves per treatment which were collected ten (10) days before harvest. The preparation of the samples in the laboratory followed the following procedure: at first, samples were oven-dried at 90 °C for 68 h. Then, they were grounded into a fine powder using a mill (pulverisette 11, Fritsch GmbH 93 Milling and Sizing, Idar-Oberstein, Germany) and were stored in falcon tubes placed in glass desiccators until IRMS analysis. Before analysis, samples were oven-dried again at 90 °C for 48 h. The results of the isotope ratio analysis were expressed in permille (‰) using the delta “δ” notation which was calculated according to the following equation:

$$\delta^{13}C\left(‰\right)=\frac{R_{\mathrm{sample}}-R_{\mathrm{standard}}}{R_{\mathrm{standard}}}\times 1000$$

where R_sample_ and R_standard_ are the ^13^C/^12^C ratio of the sample and standard (V-PDB) respectively. The results were normalized to VPDB using IAEA-600 (Caffeine, IAEA, Vienna, Austria), with assigned carbon isotope delta values and standard uncertainties (δ^13^CV-PDB = −27.77‰ ± 0.043‰) [56].

### 2.8. Statistical Analysis

Differences in all parameters between the “ON” and “OFF” study years were assessed using a T-test at a 95% confidence level after testing for normal distribution. The standard error (SE) was used to measure statistical spread. Statistical analysis was performed with SPSS v.27. The values of sap flow are represented as means of eight measurements (*n* = 8), i.e., four measurements per plant from two selected trees. Regarding the measurements of maximum photosynthetic rate (A) and stomatal conductance (g_s_), the values presented are the average of ten measurements (five measurements per tree). Water potential values measured with the pressure chamber bomb at pre-dawn represent the mean of ten measurements (five per tree), and the measurements from the psychrometry are the mean of two values (one measurement per tree).

## 3. Results

### 3.1. Environmental Parameters and Tree Growth Pattern

**Figure 1:**
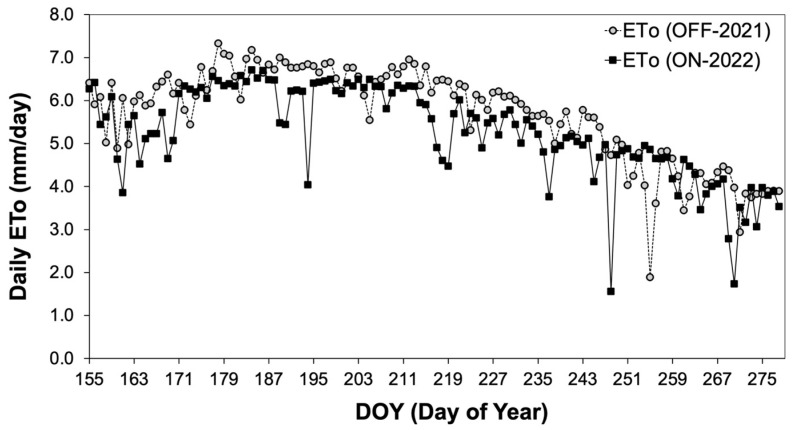
Comparative assessment of ETo during the two study years (DOY 155 = 4 June).

The environmental conditions were assessed by comparing potential evapotranspiration (ETo) during both study years. The results indicated that average ETo during the growing season of the “OFF” year (2021) exhibited significantly higher values (5.22 ± 0.13 mm/day) compared to that during the “ON” year (4.83 ± 0.12 mm/day) (Figure 1, Table 1). As far as leaf area index (LAI) is concerned, there were no significant differences between the two study years (Table 1), a fact that can be attributed to the local farmer’s cultivation practice of avoiding tree pruning after an “OFF” year in order to maintain annual shoots for fruit-bearing in the following year.

### 3.2. Soil Moisture Content and Sap Flow Dynamics

**Figure 2:**
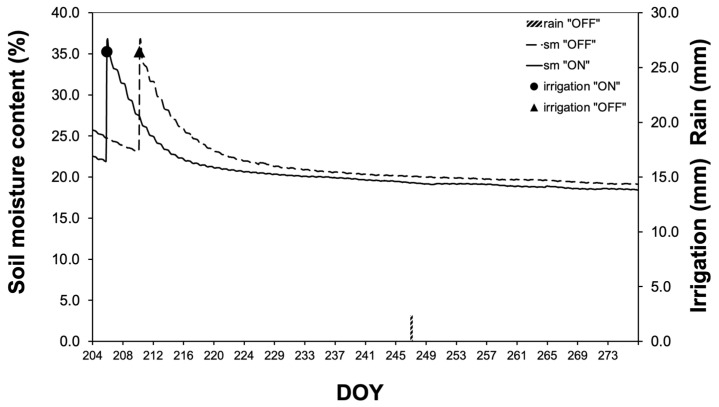
Soil moisture dynamics during the study period (DOY 204 = 23 July).

Changes in soil moisture dynamics are shown in Figure 2. Two irrigation events (one per year) at DOY 205 of the “ON” year and DOY 210 of the “OFF” year, which lasted for almost 4 h each and applied a total of 26.44 mm of water, are evident. During the growing season, no rainfall was recorded during the “ON” year, while during the “OFF” year, one rainfall of a magnitude of 3 mm occurred and is considered insignificant since it did not affect soil moisture content.

**Figure 3:**
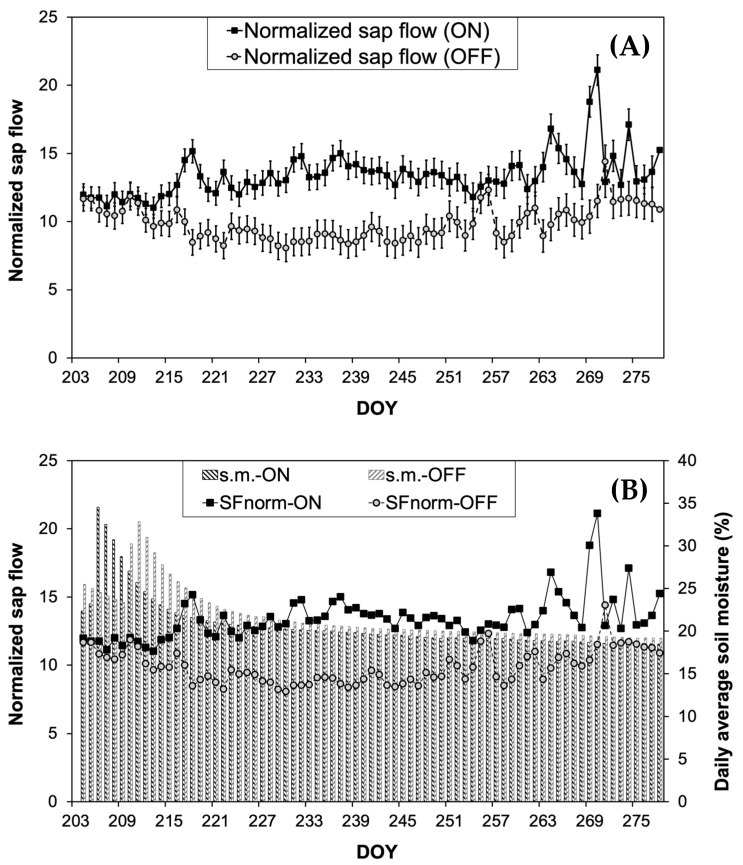
Changes in SF_norm_ (**A**) and SF_norm_ in relation to the daily average soil moisture content (s.m.) (**B**) during the experimental period.

During the major part of the growing season (DOY 214 to 270), olive plants exhibited significantly higher normalized sap flow (SF_norm_) values in the “ON” year compared to those in the “OFF” year. Despite these differences in SF_norm_, no significant differences in soil moisture content were recorded between the two study years (Figure 3B).

### 3.3. Assessment of Plant Water Status

**Figure 4:**
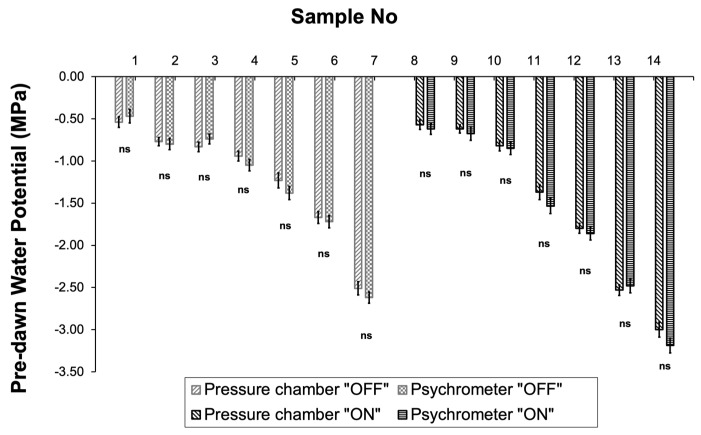
Comparison of pre-dawn water potential (Ψ_PD_) measurements obtained with the pressure chamber and the psychrometer in 14 samples. Significances: ns, no significant differences (*p* < 0.05).

Hydrodynamic measurements conducted using either the pressure chamber technique or the psychrometer resulted in almost similar values of pre-dawn leaf water potential values during the “ON” year and the “OFF” year (Figure 4).

**Figure 5:**
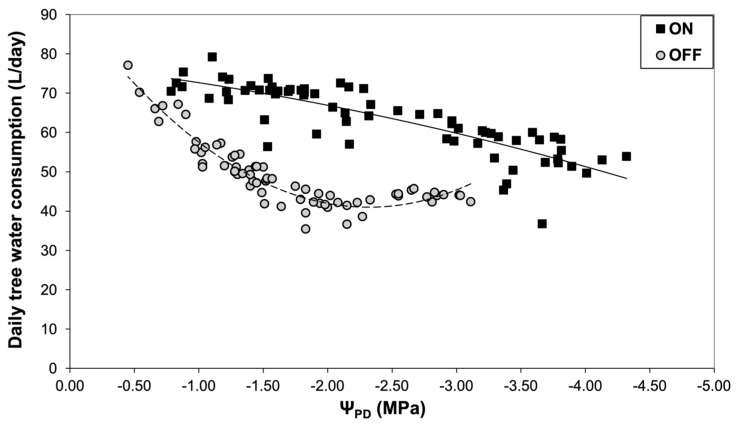
Relationship between daily water consumption (liters/day) and plant water status during the “ON” and “OFF” years.

**Figure 6:**
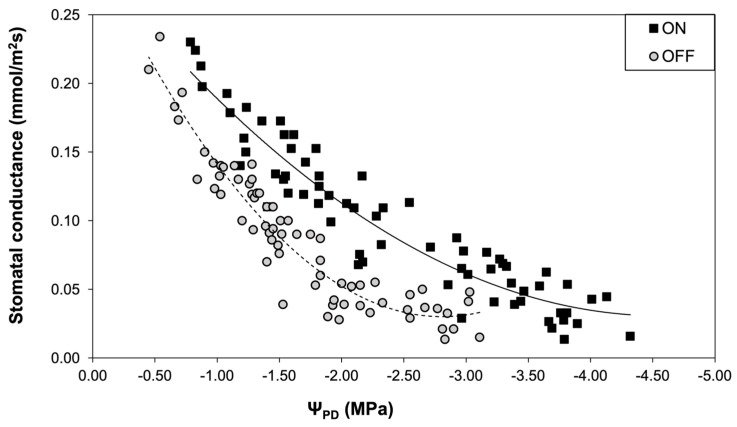
Stomatal conductance (g_s_) during two different fruiting years in relation to plant water status.

The measurements of Ψ_PD_ obtained with the psychrometers were then used to elucidate the relationship between plant water status and daily sap flow (Figure 5). The results indicated significantly higher tree water losses during the “ON” year compared to the “OFF” year under the same plant water status conditions, which could be attributed to the higher stomatal conductance (Figure 6). Indeed, olive plants exhibited higher values of maximum stomatal conductance in the “ON” years under similar plant water status conditions.

**Figure 7:**
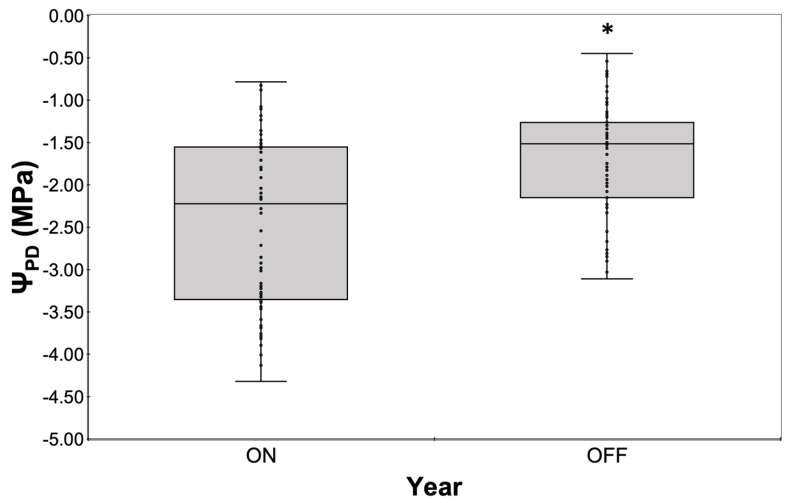
Comparative assessment of Ψ_PD_ measured with thermocouple psychrometers during the two study years. Significances: *, statistically significant differences (*p* < 0.05).

In addition, this differentiation in water consumption seemed to affect tree water status, with Ψ_PD_ being significantly higher during the “OFF” year compared to the “ON” year (Figure 7). Ψ_PD_ values during the “ON” year ranged from −0.785 MPa (measured after irrigation) to −4.32 MPa at the end of the drying cycle, while the corresponding range during the “OFF” year was from −0.45 MPa to −3.11 MPa respectively.

**Figure 8:**
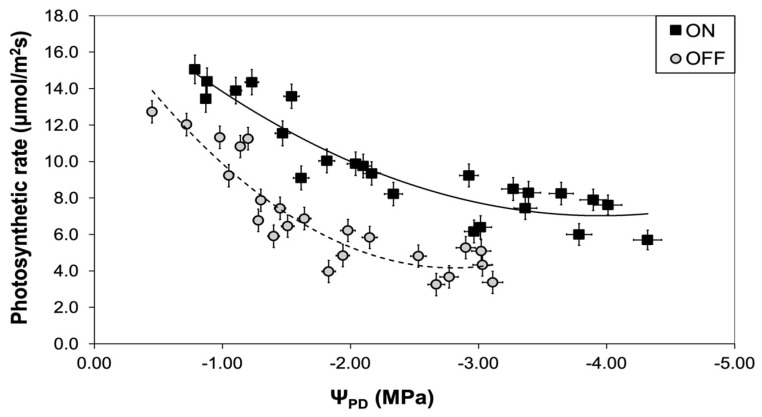
Correlation between plant water status and photosynthetic rate during “ON” and “OFF” years.

A similar pattern to the stomatal conductance was also observed concerning the photosynthetic rate. The comparison between the two experimental years revealed that the photosynthetic rate of trees in the “OFF” year was significantly lower than in the “ON” year, irrespective of the plant water status conditions (Figure 8).

**Figure 9:**
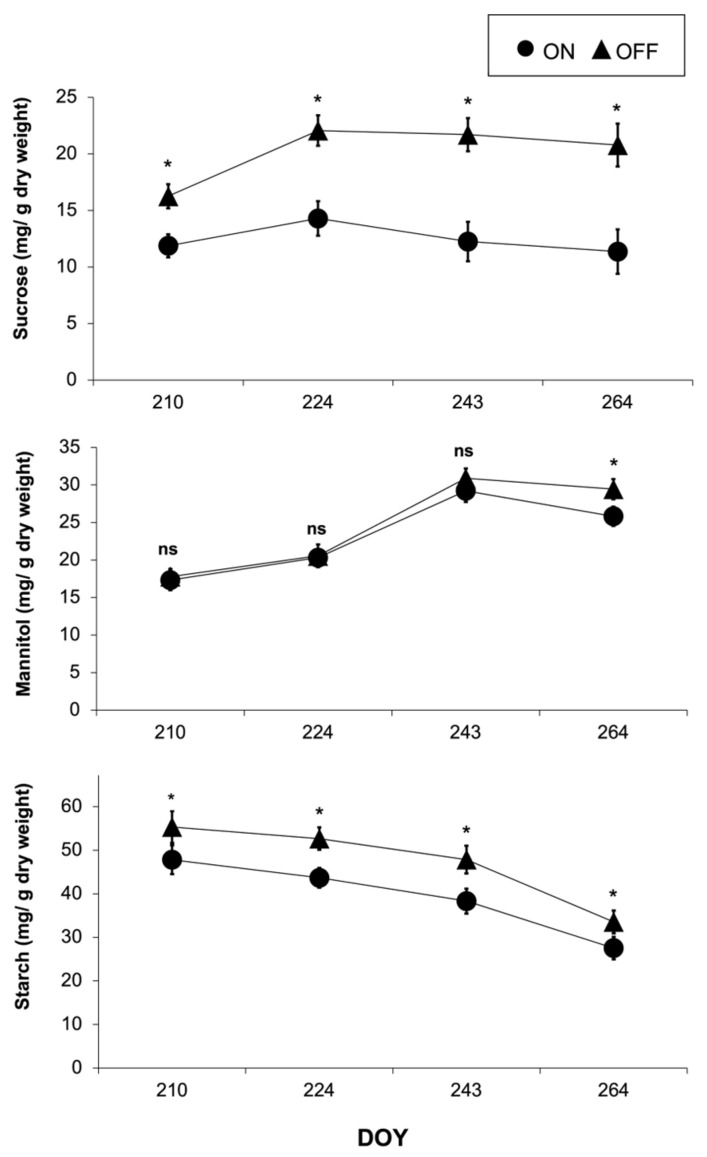
Sucrose, mannitol, and starch concentration in olive leaves during the “ON” and “OFF” years. Significances: *, statistically significant differences (*p* < 0.05), ns: no significant differences (*p* < 0.05).

The concentration of sucrose and starch varied significantly, while the concentration of mannitol showed no significant difference between the two study years (Figure 9). During the “ON” year, the concentration of mannitol decreased at the end of September, while it remained nearly constant in the “OFF” year. Starch was the most abundant among the three measured carbohydrates, with an average concentration of ca. 41% and ca. 68% higher than mannitol and sucrose, respectively.

**Figure 10:**
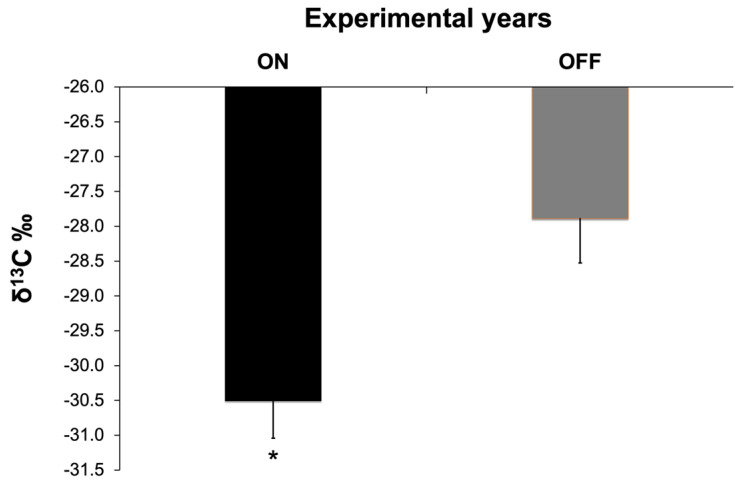
Stable carbon isotope ratio (δ^13^C) during the two study years. Significances: *, statistically significant differences (*p* < 0.05).

The carbon-stable isotope ratio also exhibited a statistically significant difference between the two study years (Figure 10). During the “OFF” year, leaves fixed a significantly higher amount of ^13^C compared to the “ON” year.

## 4. Discussion

Changes in environmental parameters indicated that the “OFF” year (from DOY 204 to DOY 278) was significantly drier compared to the corresponding period of the “ON” year (Figure 1, Table 1). Thus, it was expected that tree water consumption should be higher during the “OFF” year, leading to a higher tree water consumption rate [57]. However, this was not evident in our results. The comparison of the SF_norm_, which by definition integrates the effect of the prevailing climatic conditions, clearly shows that the presence of fruits, which are considered the most potent sinks [58], is the preponderant factor responsible for the significantly higher tree water consumption (Figure 3A). This is also supported by the fact that even under similar soil moisture conditions, SF_norm_ was significantly higher during the “ON” year (Figure 3B). A reduction in water consumption during the “OFF” year in olive trees is also reported by [59], who attributed the differences in water consumption to the substantial changes induced by different fruit loads to tree canopy size. However, in our study, the measurements of LAI indicated that canopy size remained relatively constant between the “ON” and “OFF” years, providing evidence that the sink–source effect was the determinant factor for differences in plant water consumption. Similar results regarding the sink–source effect on tree water consumption were reported in other studies related to olives [32], avocados [8], grapevines [15,60] and coffee [61], as a result of the significant increase in the stomatal conductance of heavy-fruiting trees. On the other hand, higher water consumption measured in the “ON” year trees could explain the significantly lower values of Ψ_PD_. This is also confirmed by previous findings that highlight the effect of the sink–source ratio on water potential in apples [62,63], nectarines [27], and peaches [64]. In contrast, Gucci et al. [33] reported no effect of the sink–source ratio on Ψ_PD_. However, in our results, the acquisition of a substantial amount of water potential data using the psychrometry method, which have been previously verified for their accuracy, provides a more detailed understanding of the dynamic of potential leaf water changes in response to differences in fruit load. According to this data, Ψ_PD_ during the “ON” year dropped significantly to −4.32 MPa, indicating a moderate-to-high plant stress level [65]. Despite the above-mentioned low Ψ_PD_ values, olive trees are well known for their ability not only to withstand such conditions [66], but also for preserving leaves’ physiological performance, including the production of assimilates [67,68].

The downregulation of photosynthesis, which usually occurs under low fruit-bearing conditions, has been attributed to the rapid accumulation of photosynthesis end products such as starch and soluble sugars in the leaves caused by the imbalance between the carbon assimilation rate and the carbon export rate to sinks [10,22,69,70]. According to Körner [21], the accumulation of carbohydrates in leaves is part of a “feedback” mechanism resulting in a significant decrease in photosynthetic rate. In this frame, the significant accumulation of carbohydrates, namely starch and sucrose, during the “OFF” year, which was evident in our results, seemed to contribute to the downregulation of photosynthesis. Our results demonstrate that starch was the carbohydrate with the greatest concentration, followed by mannitol and sucrose. Furthermore, the concentration of starch and sucrose exhibited significant variations between the two study years in response to different crop loads, in accordance with the findings from other studies [17,22]. Conversely, no significant differences between treatments were observed regarding mannitol concentration. A possible explanation might be based on the well-known positive role of this specific carbohydrate in olive responses to abiotic stresses [71] mainly on the drought tolerance of this species [24]. In the present study, the non-significant differentiation between the study years might be attributed to the fact that during the “ON” year, the trees experienced more intense water stress conditions. Given the significant osmoregulatory role of this molecule [72], it seems possible that olive trees in the “ON” year actively accumulate mannitol in leaf cells, thus increasing their concentration. As a result, the concentration of mannitol during the “ON” year increased, reaching almost similar values to those of the “OFF” year in which the high accumulation of this carbohydrate occurred due to the absence of strong sinks. On the other hand, several studies highlighted the role of mannitol in photosynthesis as molecules facilitating carbon transport [73,74]. Indeed, in some plants, especially in brown algae and certain higher plants, mannitol serves as a substance transporting carbon dioxide between different cells or cell structures, thus maintaining the gas balance in the plant, supporting the transport and access of carbon dioxide to the sites where photosynthesis occurs [75,76]. However, the fact that no significant differences in mannitol concentration between the “ON” and “OFF” years occurred during most of the growing season, despite the observed differences in photosynthetic rate between the two treatments (Figure 8), provides no evidence of the specific role of mannitol in the process of photosynthesis.

Despite numerous studies, the mechanisms and interspecific differences of the downregulation of photosynthesis in relation to changes in fruit load have not yet been fully understood [20,77]. However, most studies suggest that when non-structural carbohydrates accumulate in source leaves, the downregulation of photosynthesis could result not only from decreases in the content and activity of Rubisco [78], but also from anatomical factors, such as an increase in cell wall thickness [79,80], which in turn could affect mesophyll conductance leading to reduced chloroplast CO_2_ concentrations. In this frame, stable carbon isotope analysis could be a useful tool to evaluate possible alterations induced by different fruit loads to the CO_2_ resistances (stomatal and mesophyll) from the atmosphere to the sites of carboxylation. Under optimal conditions, C_3_ plants discriminate against the heavier ^13^C atom [81,82], leading to lower values of δ^13^C. In our results, the significant increase in δ^13^C values observed during the “OFF” year indicates a reduction in carbon isotope discrimination due to significant alterations in CO_2_ conductances to the sites of carboxylation. Similar results were also reported in coffee plants [61], Phaseolus vulgaris plants [78], and apple trees [83], providing evidence that alterations in both stomatal and mesophyll conductances could contribute to photosynthesis downregulation in response to changes in olive fruit load.

## 5. Conclusions

Alternate bearing significantly affects the physiological parameters of olive trees, resulting in a substantial decrease in plant water usage during periods of lower fruit load due to the sink–source effect. Consequently, plant water status is affected, leading to significantly lower water potential values during the “ON” year. The present study provides evidence that the reduction in photosynthesis in the absence of fruits was caused by the concurrent effect of carbohydrate accumulation in the leaves and stomatal closure, leading to increased total resistances in the CO_2_ pathway from the atmosphere to the chloroplasts’ carboxylation sites. Given the substantial influence of alternate bearing on hydrodynamic and ecophysiological parameters, particularly on tree water consumption, the data obtained could be very useful for the optimization of irrigation scheduling in olive orchards.

## Figures and Tables

**Table 1:** Fruit production, leaf area index (LAI), and average ET_o_ during the study years 2021 (“OFF”) and 2022 (“ON”).

Experimental Year	Fruit Production (kg/Hectare)	LAI	Average ET_o_ (mm) from DOY 204 to DOY 278
2021	3430 ± 158	2.80	5.22 ± 0.13
2022	20,400 ± 325 *	2.82 ^ns^	4.83 ± 0.12 *

Significances: *, statistically significant differences (*p* < 0.05), ns: no significant differences (*p* < 0.05).

## Data Availability

Data are available upon communication with the corresponding author.
